# Supplementary material for: Distinct basal ganglia contributions to learning from implicit and explicit value signals in perceptual decision-making
Source: Nat Commun. 2024 Jun 22;15:5317. doi: 10.1038/s41467-024-49538-w (PMC11193814; doi:10.1038/s41467-024-49538-w)
Supplement: Supplementary file 1 — Supplementary Information [file 41467_2024_49538_MOESM1_ESM.pdf]

Supplementary Figures for:

Distinct basal ganglia contributions to learning from implicit and explicit value signals in perceptual decision-making

Tarryn Balsdon[1,2*], M. Andrea Pisauro[1,3], & Marios G. Philiastides[1]

1. Centre for Cognitive Neuroimaging, School of Psychology and Neuroscience, University of Glasgow, Glasgow, UK
2. Laboratory of Perceptual Systems, DEC, ENS, PSL University, CNRS UMR 8248, Paris, France
3. School of Psychology, University of Plymouth, Plymouth, UK

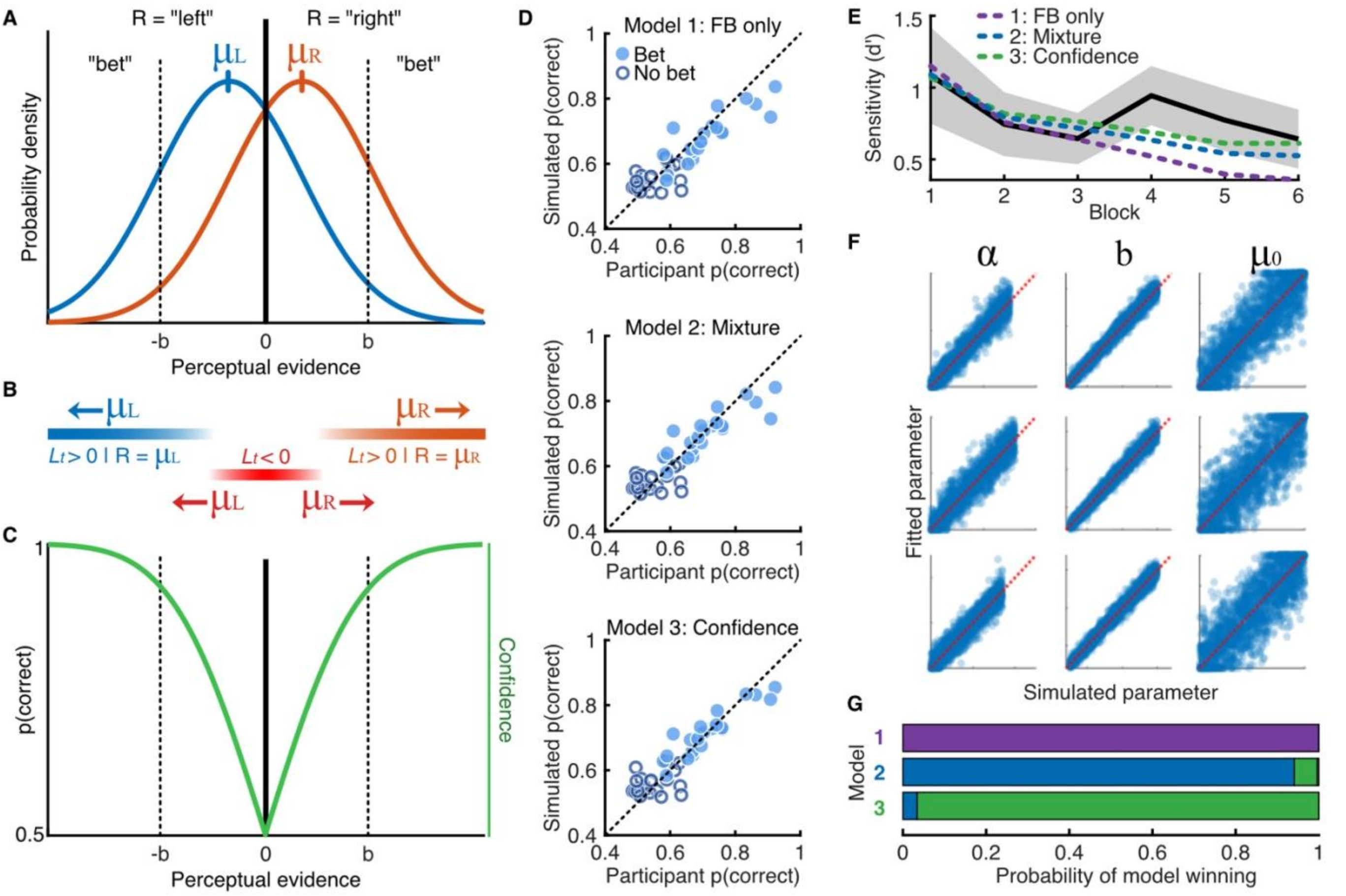

***Supplementary Figure S1. Computational modelling**. **A**) We implement a simple Signal Detection Theory framework to describe perceptual decisions and bet responses in the experiment. According to the assumptions of Signal Detection Theory, the presentation of an external stimulus results in some internal perceptual evidence which is disrupted by Gaussian distributed noise. In this case, the leftward and rightward stimuli produce perceptual evidence of different means (μ_L, and μ_R), but enough noise that the distributions of evidence overlap, meaning that on some trials a leftward stimulus results in perceptual evidence that is more similar to that resulting from a rightward stimulus. To make a decision, the observer implements a criterion, which we assume is unbiased (0) for simplicity (thick vertical line). The observer responds 'right' if the evidence exceeds this criterion. Assuming the Gaussian distributed noise has unit variance (for simplicity), the distance between the means determines the observers' sensitivity in discriminating the stimuli. Bet responses are made based on an additional criterion (b, dashed vertical lines), where the observer bets if the evidence exceeds this criterion. **B**) Previous work has demonstrates that perceptual learning increases sensitivity (equivalent to increasing the distance between the means), and this can be achieved by adjusting the weighted integration of neural activity from neurons tuned to different motion directions. In essence, a negative prediction error ($L_t < 0$, which is most likely occur from evidence close to the criterion, red bar) should prompt the observer to place less weight on neurons promoting this evidence (those tuned away from the left/right directions), and this would be equivalent to both means being shifted away from the criterion. A positive prediction error should prompt the observer to increase the weights on the evidence that led to that perceptual evidence (those tuned close to the direction of the response, blue and orange bars) and this would be equivalent to the mean for the chosen response shifting further from the criterion. **C**) Proportion correct as a function of the perceptual evidence given the distributions in A. For simplicity, we assumed confidence scaled with this ideal, and calculated trial-wise confidence from the expected value of the perceptual evidence given the distributions, the participant's perceptual decision, and bet response (which approximates the location of the evidence and its likelihood). **D**) Participant proportion correct vs the simulated proportion correct from the fitted parameters of the feedback only model (top), mixture model (middle) and the confidence model (bottom) for bet (light blue filled markers) and no-bet (dark blue open markers) separately. The model was fit to minimise the negative log-likelihood of the participants' perceptual decisions and bet responses and so gives a representation of the fit of the model to individual data. **E**) Sensitivity (d') by experimental block for participants (black, 95% within subject confidence intervals shaded) and the model predictions (dashed lines). **F**) Parameter recovery analysis: for each model, we simulated behaviour in our experiment (300 trials) and compared the simulated parameters with those fitted by our model fitting procedure. The simulated and fitted parameters showed strong linear correlation (average spearman's rho = 0.93, minimum rho = 0.85). The sum of squared error between the simulated and fitted*

parameters was on average just 15% of the squared error of the simulated parameters from their mean (maximum = 33%, for starting mu). ***G****) Model recovery analysis: we simulated behaviour from each model and fit all three models, allowing us to compare the fit across 23 participants as in our experiment. The horizontal bars show the probability of each model appearing as the winning model. If the true model was the feedback only model (model 1) the chance finding the other models superior was $p < 0.001$; for the mixture model (model 2) the chance of finding the feedback model superior was $p = 0.004$ and the confidence model, $p = 0.036$; and for the confidence model (model 3) the chance of finding the feedback model superior was $p < 0.001$ and the mixture model, $p = 0.046$. N=23 participants.*

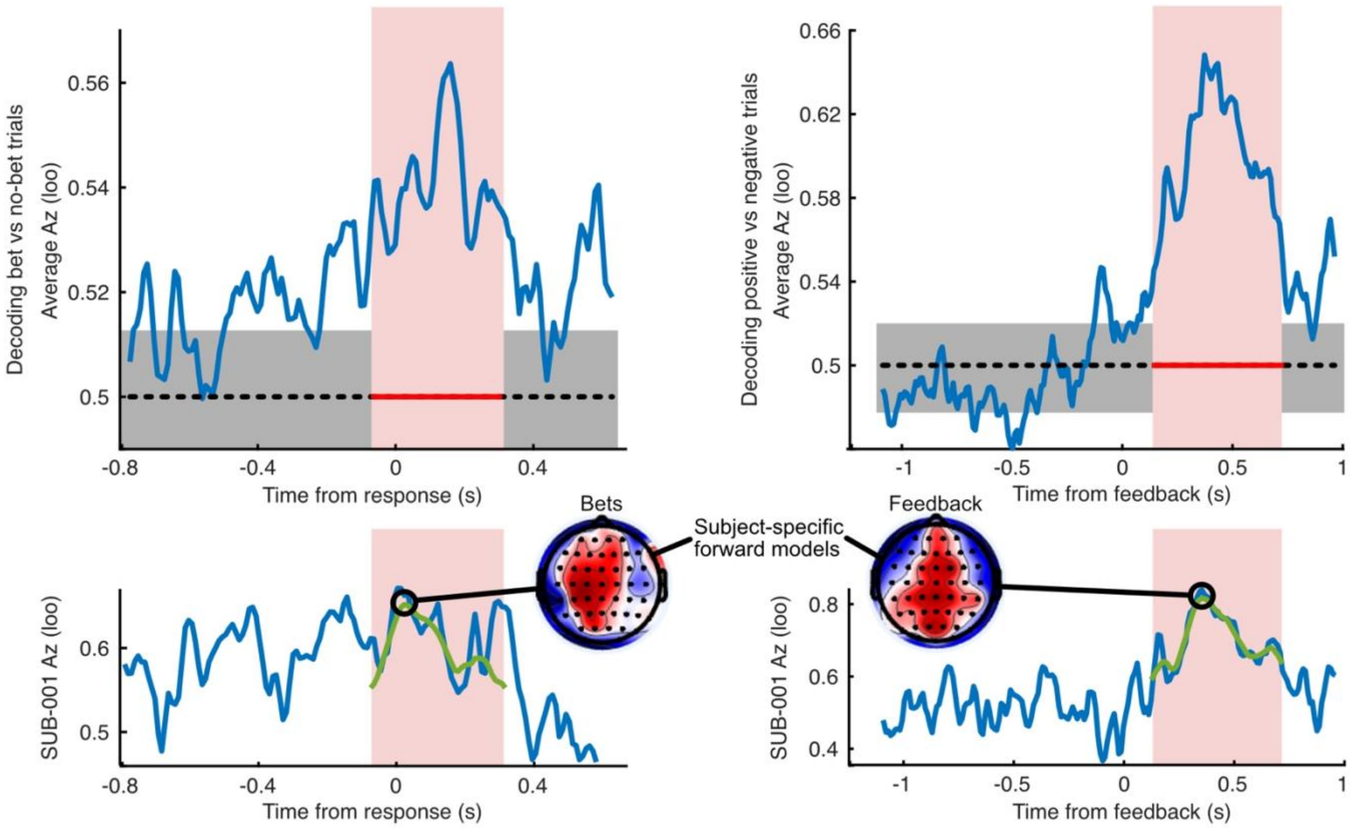

Supplementary Figure S2. *Procedure for isolating subject-specific spatial filters related to representations of confidence and explicit feedback. For each subject, we ran a linear discriminant analysis (LDA) of bet vs no-bet trials in the decision-window and positive vs negative feedback in the feedback window. The analysis was run on data within a sliding window and assessed across time using the area under the ROC (Az) in a leave-one-out (loo) validation procedure. Notably, the performance of the decoder in bet vs no-bet trials is not as high as in positive vs negative feedback, but in both cases accuracy reaches levels well above chance and is in line with previous findings (Desender et al., 2019; Gherman & Philiastides, 2018; Fouragnan et al., 2015). We suggest the decoding accuracy of confidence is less than that of explicit feedback because the representation of explicit value is more distributed across cortical sources, and perhaps the implicit representation is less variable with respect to the external variable (for confidence there is more time in which the representation could evolve prior to the bet response). We assessed the group-level significant time window in each case (red shaded areas), and then took the best time-point for individual subjects from a 9-point moving average within this window (green line). The topographies show the resulting forward models (scalp projections of the spatial filter) at this time. The analysis in the Manuscript Figures 2A and 2B is performed by applying these spatial filters over time to generate the predicted value (bet-prediction, or feedback-prediction), for each trial, and then averaging across trials, taking either bet and no-bet trials separately, or explicit feedback trials separately. N=23 participants.*

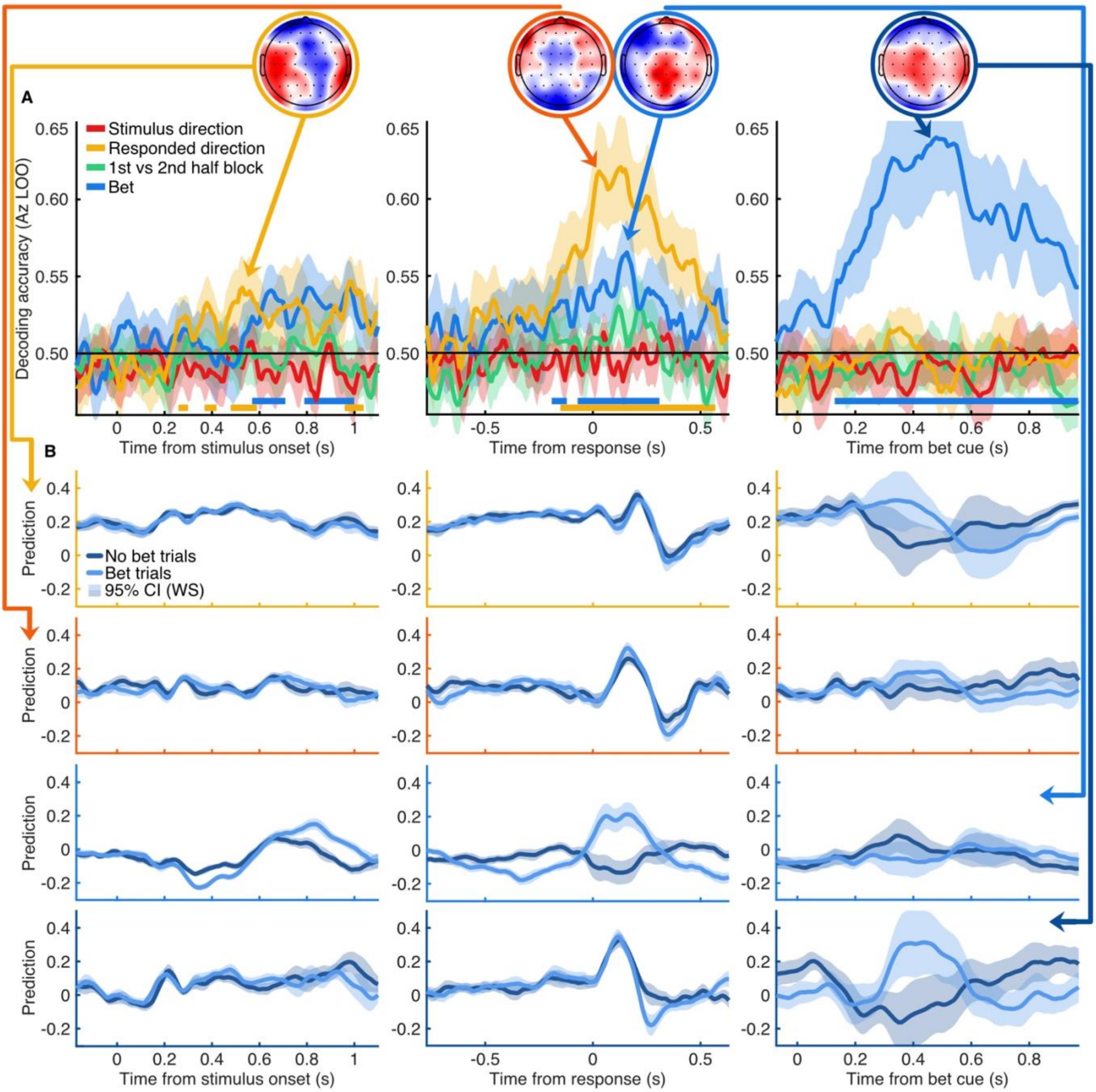

Supplementary Figure S3. Decoding of variables around the decision. A*)* *Decoding accuracy (area under the ROC (Az) in a leave-one-out validation procedure) for epochs of EEG data locked to stimulus onset (left) the perceptual decision response (middle) and the cue to place a bet (right). Horizontal black line shows theoretical chance (0.5). We attempted to decode the presented stimulus direction (left vs right, red); the reported direction of the perceptual decision response (yellow), trials in the 1st vs 2nd half of each block (to target learning to improve sensitivity over the course of the block, green); and bet vs no-bet responses (blue). Horizontal coloured bars show significantly above chance decoding performance (corresponding colours) based on t-tests against theoretical chance. We did not observe above chance decoding for stimulus direction nor for trials in the 1st vs 2nd half of the block. We observed significant decoding performance for the responded direction in stimulus onset epochs and response epochs. There were windows of significant decoding of bet responses in all epochs. The distinct topographies (above) suggest these decoders were relying on distinct sources of EEG activity.* ***B****)* *Generalisation of the spatial filters over time. As for the bet- and feedback-predictions in the main manuscript, we selected spatial filters for each participant for each of the highlighted significant windows (corresponding to the topographies) and applied these filters over time within each of the epochs. Axis colours and arrows highlight which row corresponds to which decoding analysis: decoding the responded direction in the stimulus epochs (top), in the response epoch (second); decoding bets in the response epochs (third – this is the bet-prediction in the manuscript) and the bet-cue epochs (bottom). The y-axis corresponds to the prediction in terms of the decoded variable, but split by bet and no-bet trials. Decoding the responded direction shows no sign of discriminating bet from no-bet trials, indicating the EEG activity relevant for decoding the responded*

direction could not be contaminating the bet-prediction. The decoder discriminating bets in the response-epochs does not generalise to the bet-epochs, in a double-dissociation with the decoder discriminating bets in the bet-epochs which does not generalise to the response-epochs. This is important because the bet-epochs contain the finger movements which provide clear EEG activity to discriminate bet from no-bet trials, yet has little to do with post-decision confidence. The double dissociation suggests EEG activity related to finger movements did not contaminate the post-decision bet-prediction. N=23 participants.

Stimulus Onset: EEG-informed fMRI Analysis

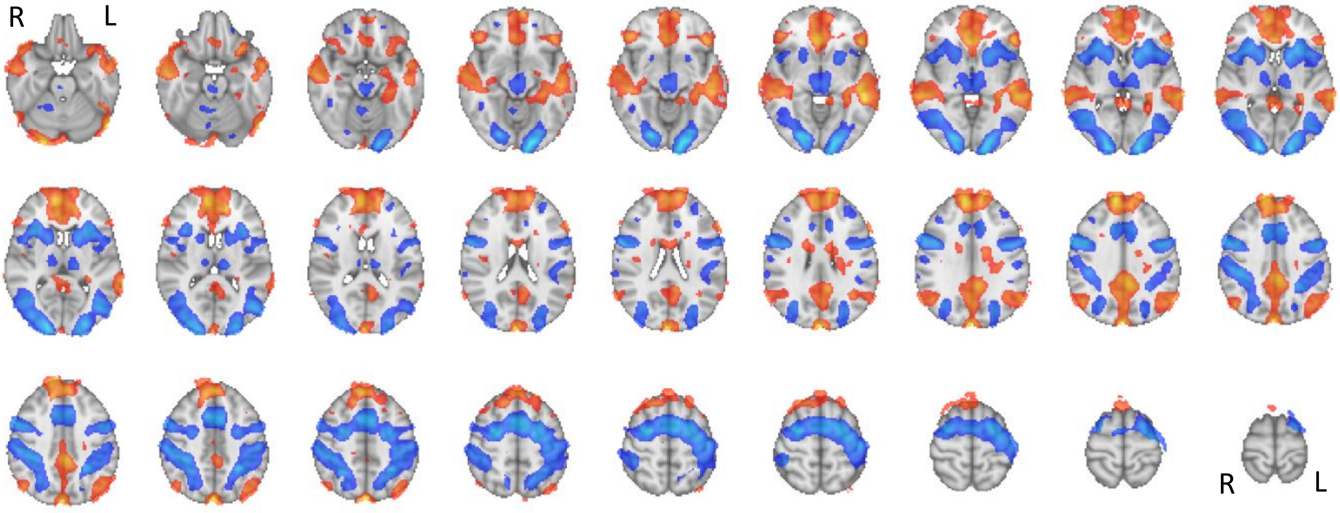

Stimulus Onset: Stand-alone fMRI version

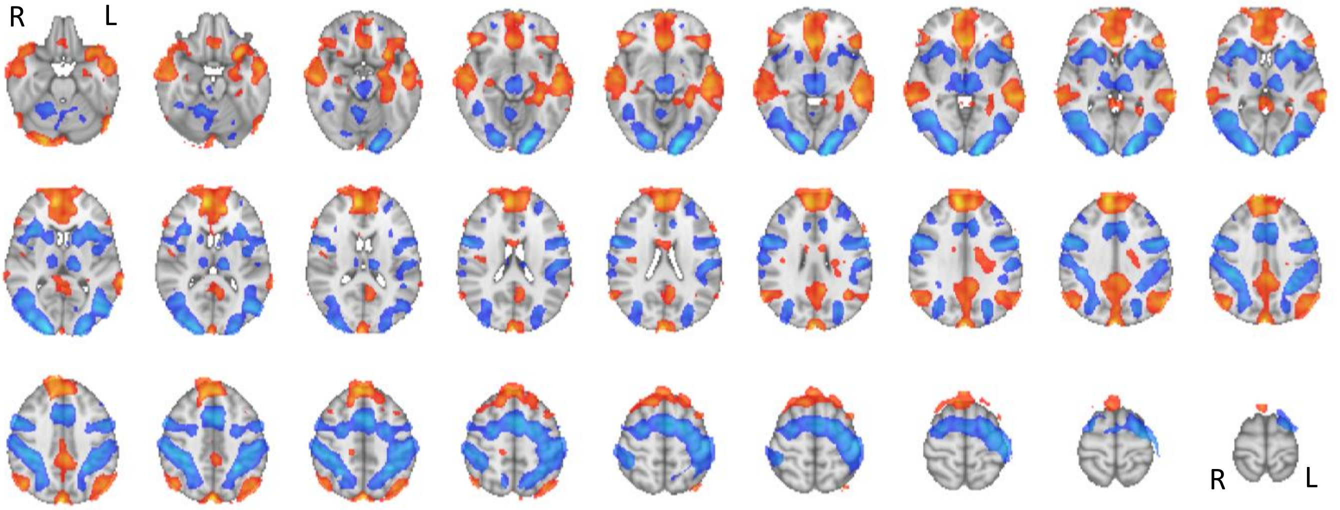

Supplementary Figure S4. *Stimulus onset Z-statistics for the EEG-informed fMRI analysis and the stand-alone fMRI version. The stimulus onset regressor was a boxcar function with duration 0.1 and amplitude modulated by the relative response time of the perceptual decision. Red shows Z-statistics <= -2.57 and Blue, >= 2.57. No minimum cluster size was applied in this display. These two analyses produce very similar results because the stimulus onset regressor was the same across the two analyses. Moreover, the different regressors that follow did not influence these results. N=23 participants.*

Post decision confidence: EEG-informed fMRI Analysis

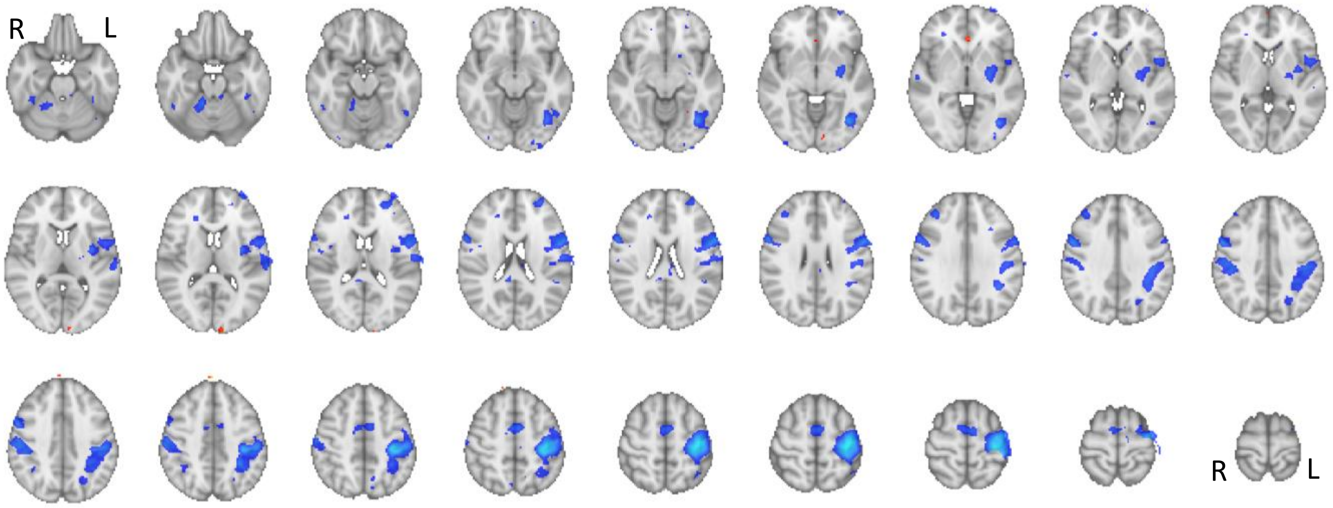

Post decision confidence: Stand-alone fMRI version

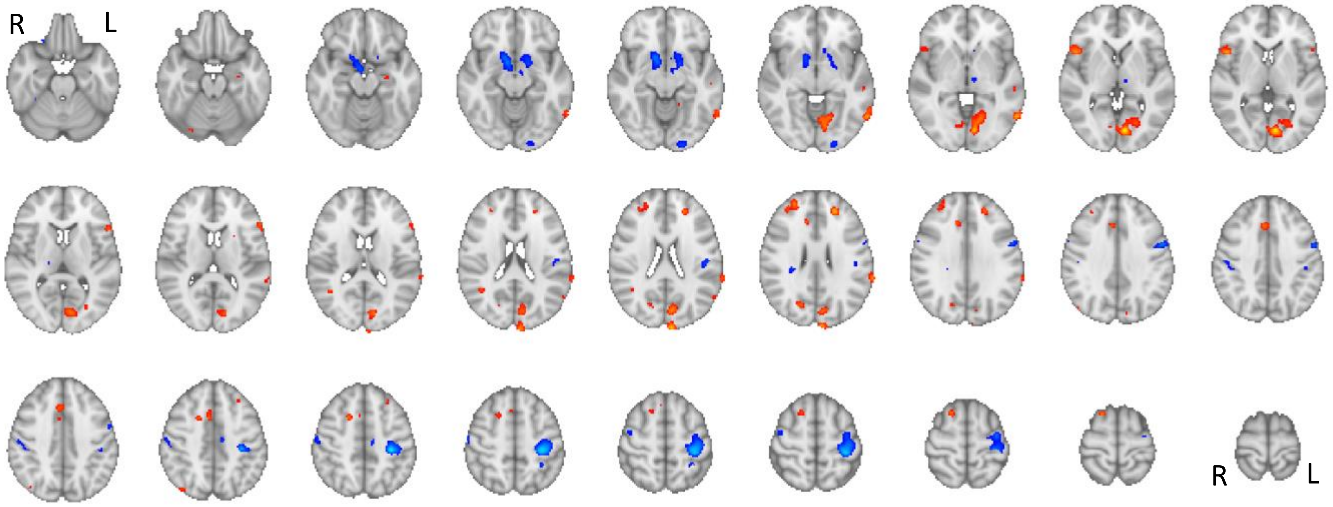

Supplementary Figure S5. *Post-decision confidence Z-statistics for the EEG-informed fMRI analysis (EEG bet-prediction following the perceptual decision) and the stand-alone fMRI version (binary bet = 1, no-bet = -1 following the decision). The regressor was a boxcar function with duration 0.1 and amplitude modulated by the EEG bet-prediction following the decision (or the binary behavioural variable for the stand-alone version). Red shows Z-statistics <= -2.57 and Blue, >= 2.57. No minimum cluster size was applied in this display. Here the EEG-bet prediction shows positive BOLD activations more strongly in IFG, GPe, prefrontal cortex and motor/somatosensory cortex. The stronger activation over motor/somatosensory cortex is curious, since the stimulus onset regressor should have also captured differences due to reaction time, and the stand-alone bet-regressor is directly related to the future bet button press and so would have resulted stronger BOLD this were due to preparatory motor activity. Also of interest is the negative BOLD correlations present in the stand-alone analysis, which are absent from the EEG-informed analysis. The largest of these clusters is in occipital cortex, and so could be related to the shorter duration from stimulus offset to the response cue on high confidence trials. We suggest this is indicative of the more fine-tuned and specific nature of the EEG-informed analysis. N=23 participants.*

Expected outcome value: EEG-informed fMRI Analysis

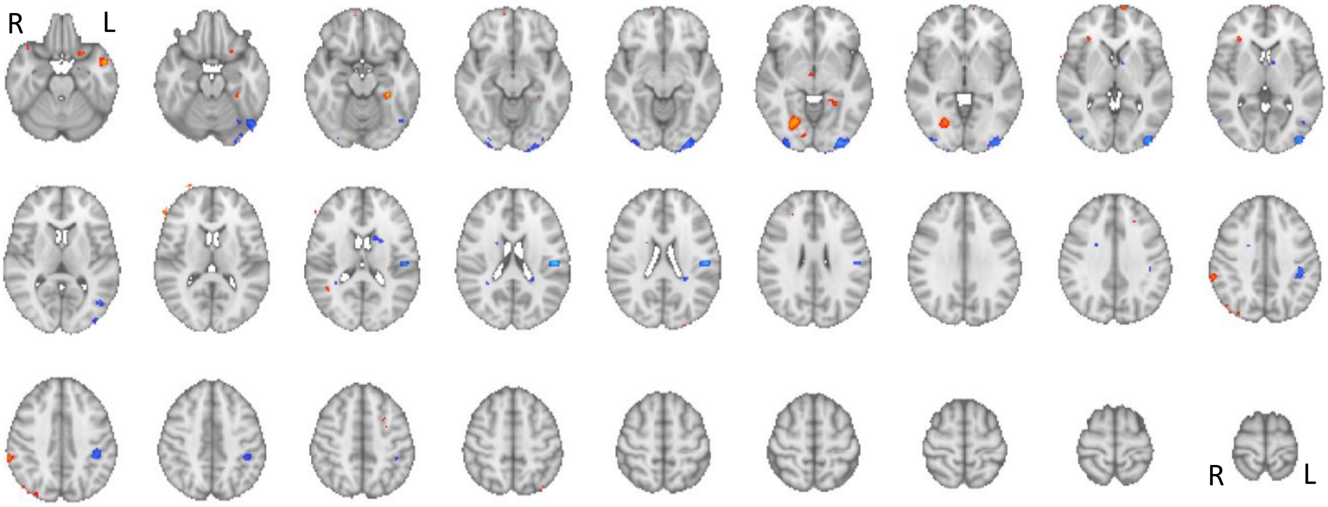

Supplementary Figure S6. *Expected outcome value Z-statistics for the EEG-informed fMRI analysis (EEG feedback-prediction in the decision-window). There is no behavioural proxy for this variable for a stand-alone fMRI analysis. The regressor was a boxcar function with duration 0.1 and amplitude modulated by the EEG feedback-prediction following the decision. Red shows Z-statistics <= -2.57 and Blue, >= 2.57. No minimum cluster size was applied in this display. N=23 participants.*

Bet cue on bet trials: EEG-informed fMRI Analysis

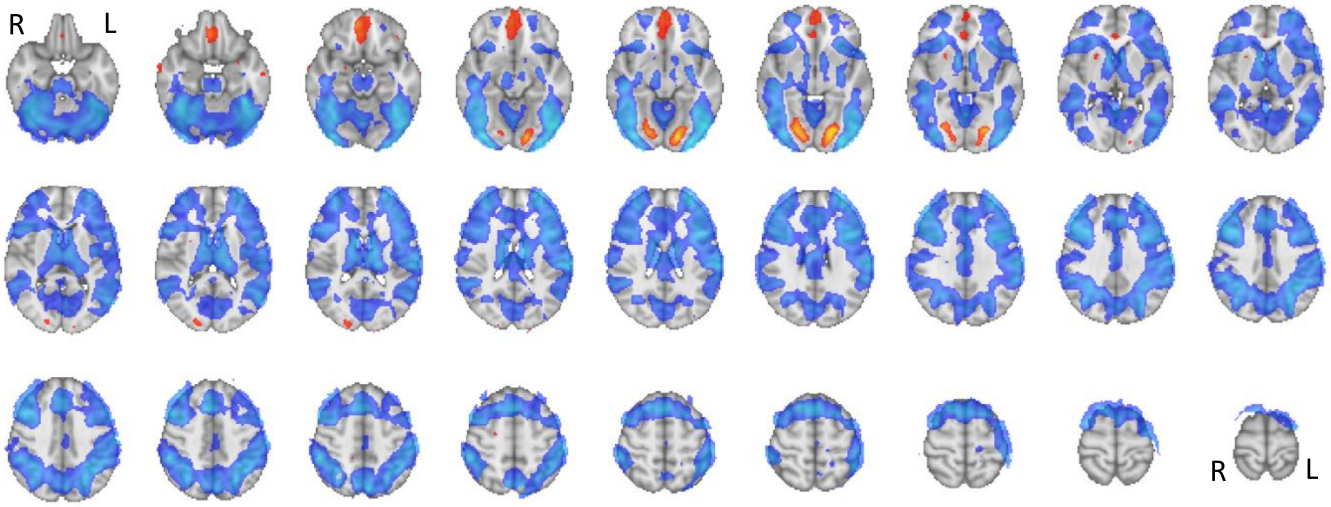

Bet cue on bet trials: Stand-alone fMRI version

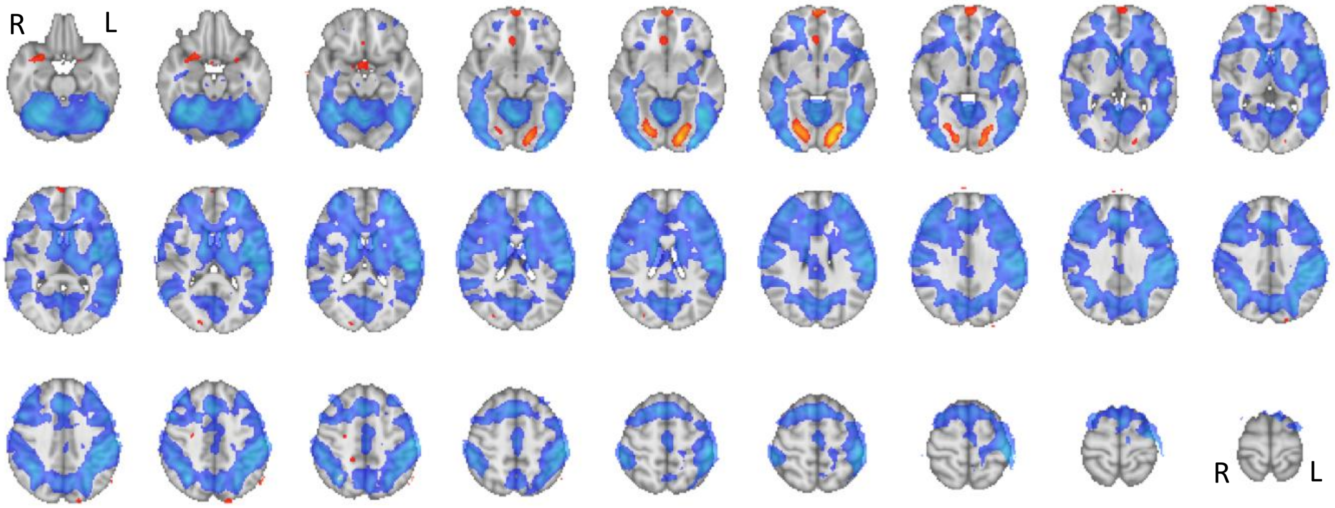

Supplementary Figure S7. *Bet Cue (on bet trials) Z-statistics for the EEG-informed fMRI analysis and the stand-alone fMRI version. The stimulus onset regressor was a boxcar function with duration equal to the bet response time and unmodulated amplitude. Red shows Z-statistics <= -2.57 and Blue, >= 2.57. No minimum cluster size was applied in this display. These two analyses produce very similar results because the stimulus onset regressor was the same across the two analyses. Moreover, the different regressors that precede, and follow, did not influence these results. N=23 participants.*

Bet cue on no-bet trials: EEG-informed fMRI Analysis

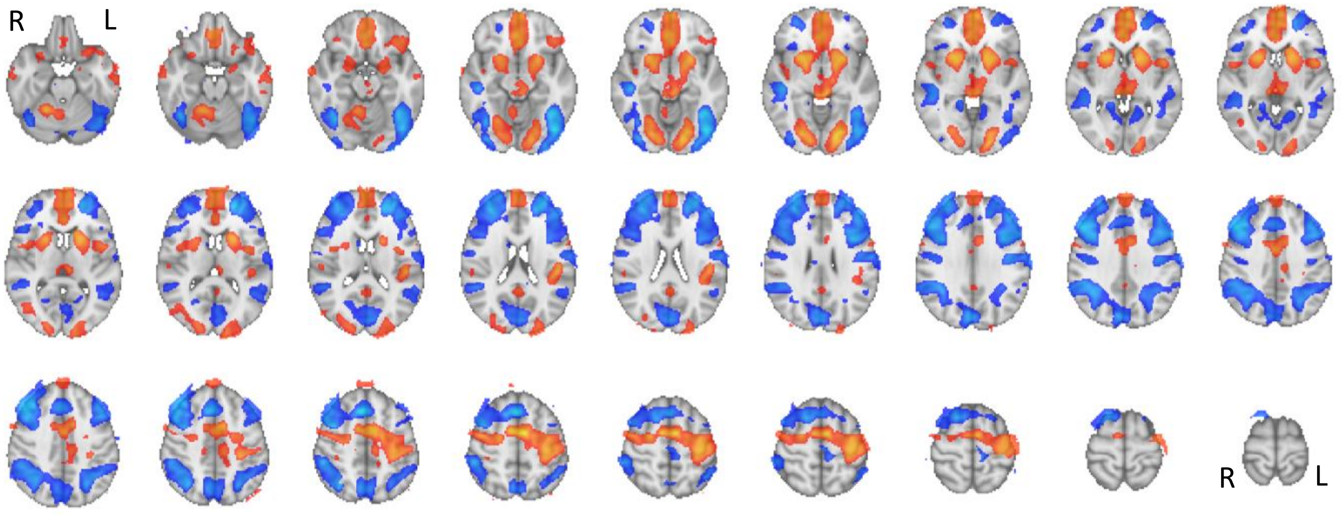

Bet cue on no-bet trials: Stand-alone fMRI version

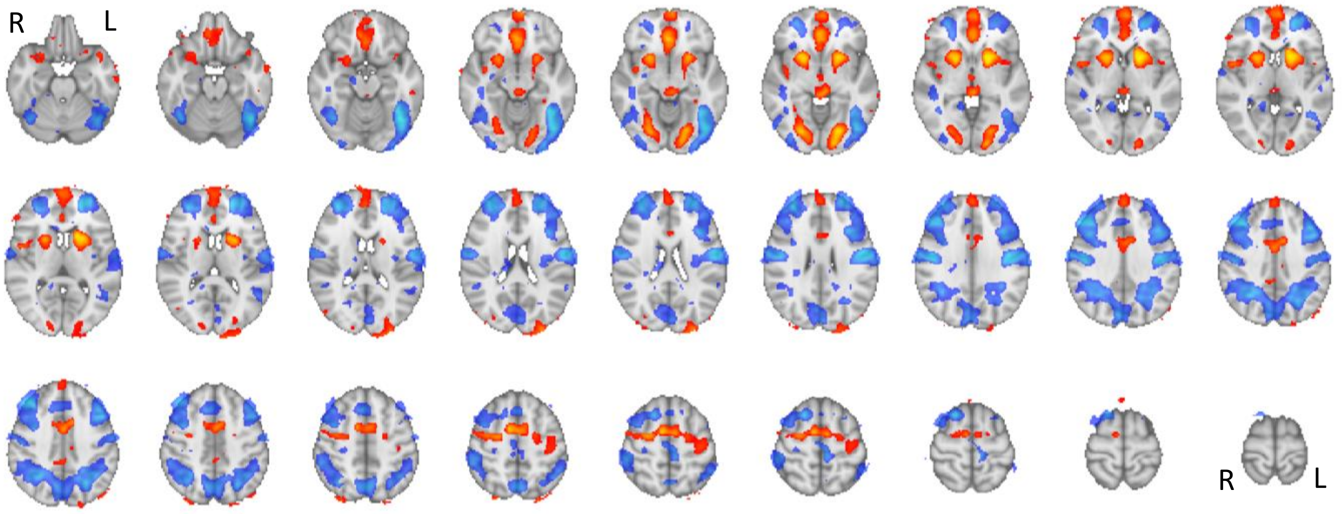

Supplementary Figure S8. *Bet Cue (on no-bet trials) Z-statistics for the EEG-informed fMRI analysis and the stand-alone fMRI version. The stimulus onset regressor was a boxcar function with duration 0.1 and unmodulated amplitude. Red shows Z-statistics <= -2.57 and Blue, >= 2.57. No minimum cluster size was applied in this display. These two analyses produce very similar results because the stimulus onset regressor was the same across the two analyses. Moreover, the different regressors that precede, and follow, did not influence these results. N=23 participants.*

Feedback cue: EEG-informed fMRI Analysis

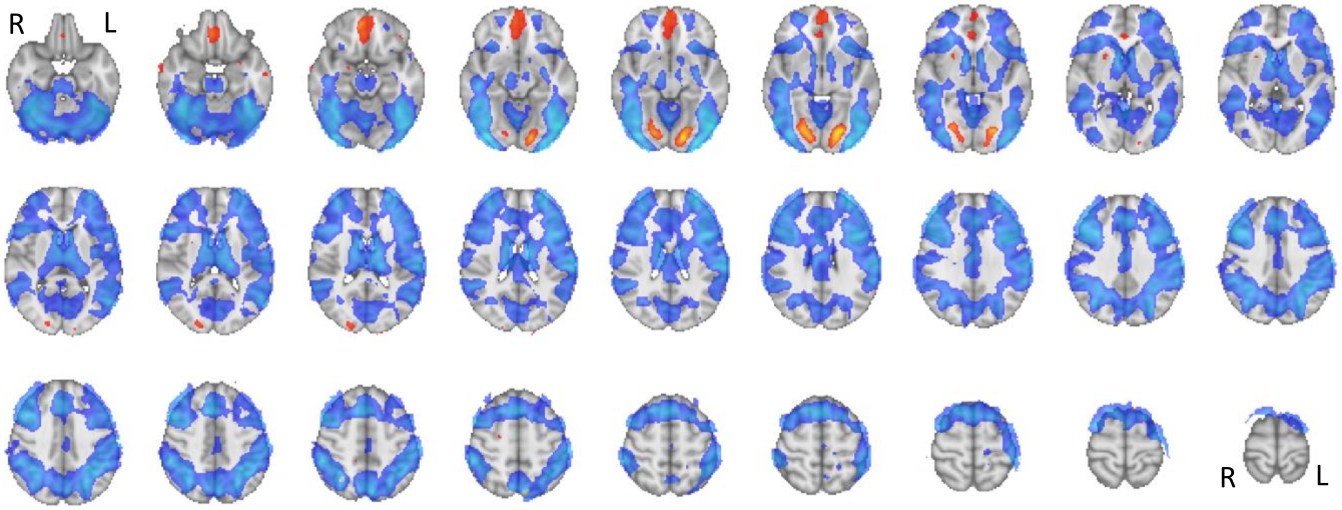

Feedback cue: Stand-alone fMRI version

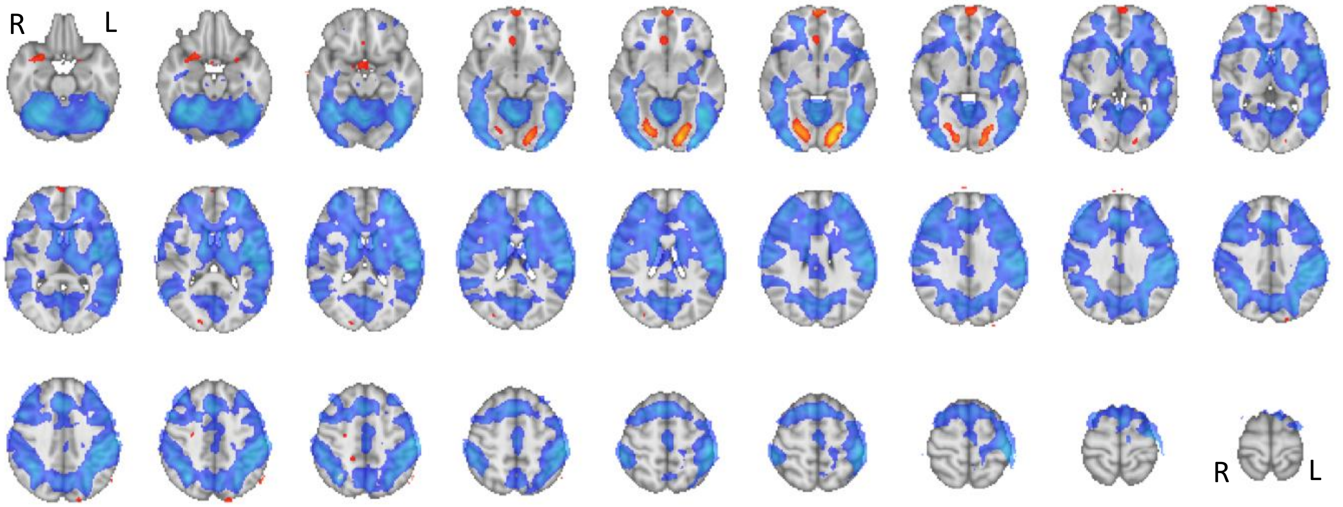

Supplementary Figure S9. *Feedback Cue Z-statistics for the EEG-informed fMRI analysis and the stand-alone fMRI version. The stimulus onset regressor was a boxcar function with duration 0.1 and unmodulated amplitude. Red shows Z-statistics <= -2.57 and Blue, >= 2.57. No minimum cluster size was applied in this display. These two analyses produce very similar results because the stimulus onset regressor was the same across the two analyses. Moreover, the different regressors that precede, and follow, did not influence these results. N=23 participants.*

Explicit outcome value: EEG-informed fMRI Analysis

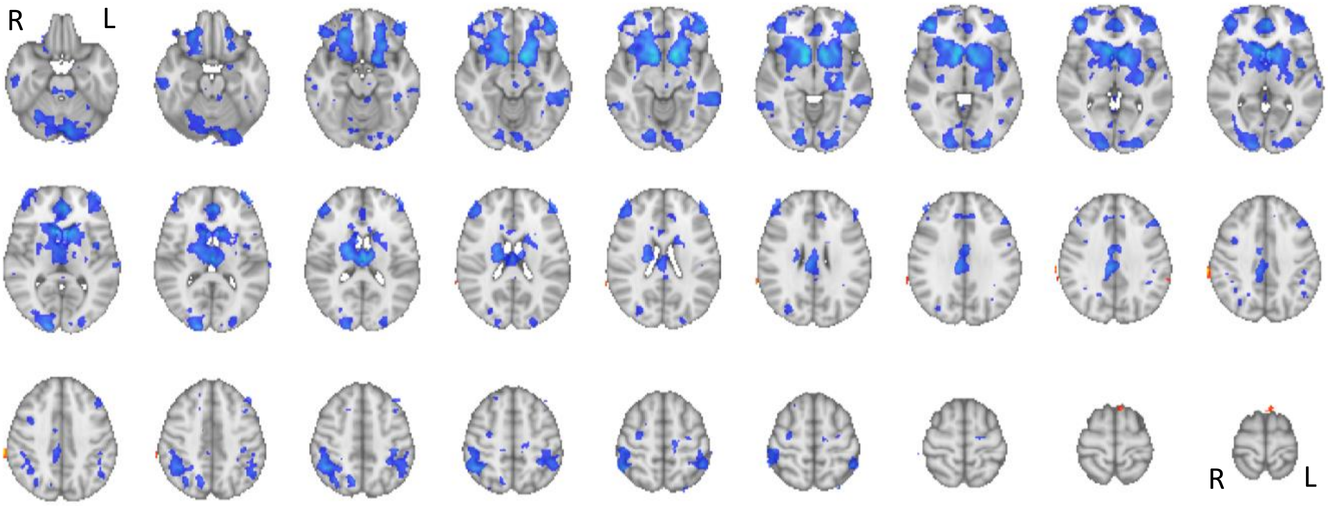

Explicit outcome value: Stand-alone fMRI version

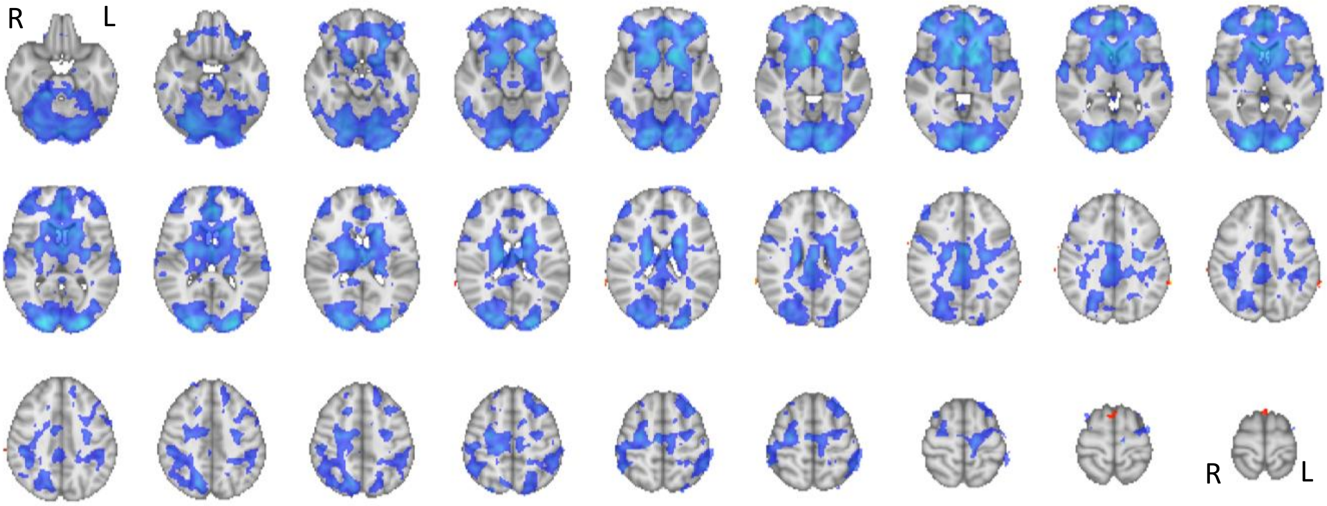

Supplementary Figure S10. *Explicit outcome value Z-statistics for the EEG-informed fMRI analysis (EEG feedback-prediction following explicit feedback) and the stand-alone fMRI version (explicit feedback signed value). The regressor was a boxcar function with duration 0.1 and amplitude modulated by the EEG feedback-prediction following feedback (or the explicit feedback signed value for the stand-alone version). Red shows Z-statistics <= -2.57 and Blue, >= 2.57. No minimum cluster size was applied in this display. The difference in these two analyses is again suggestive of the more specific nature of the EEG-informed analysis. While frontal and subcortical regions typically associated with feedback and value emerge from both analyses, the occipital regions are much less prevalent in the EEG-informed analysis. The greater luminance for positive feedback (due to the + vs – sign on the screen) means that visual cortical activity does correspond to the stand-alone positive vs negative feedback regressor, but is much less correlated with the graded EEG-informed regressor. That the frontal and subcortical regions remain strong is indicative of the fact that the graded EEG regressor does align with the endogenous variability in the graded neural representation of outcome value. N=23 participants.*

Implicit outcome value: EEG-informed fMRI Analysis

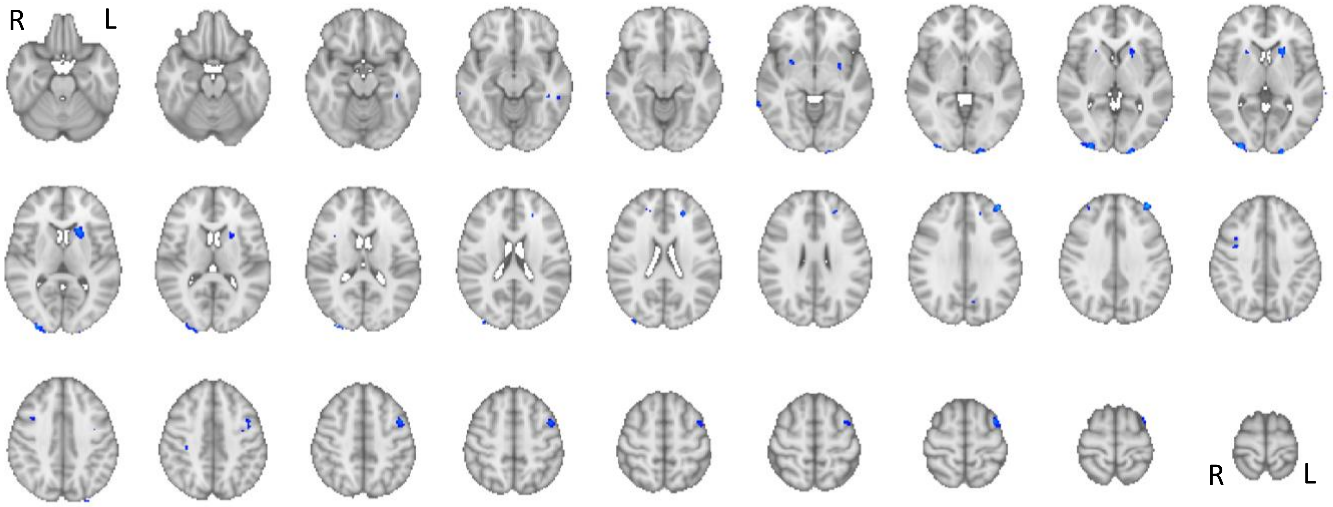

Implicit outcome value: Stand-alone fMRI version

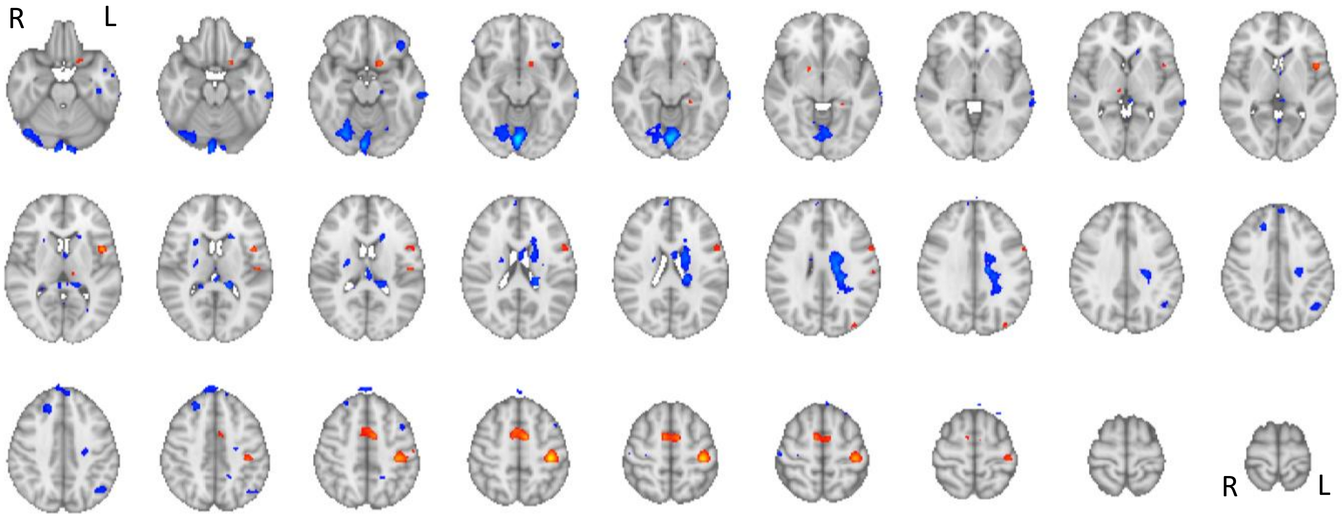

Supplementary Figure S11. *Implicit outcome value Z-statistics for the EEG-informed fMRI analysis (EEG bet-prediction in the feedback-window, on no-feedback trials, top) and the stand-alone fMRI version (binary bet = 1, no-bet = -1 following no-feedback cue, bottom). The regressor was a boxcar function with duration 0.1 and amplitude modulated by the EEG bet-prediction following feedback (or the behavioural variable for the stand-alone version). Red shows Z-statistics <= -2.57 and Blue, >= 2.57. No minimum cluster size was applied in this display. Here the EEG-informed analysis is much more specific, with three clusters surviving correction: right occipital, left middle frontal gyrus and left dorsal striatum. The left dorsal striatum cluster was not significant in the stand-alone analysis. The stand-alone analysis does show a cluster that overlaps with left caudate, but much of this cluster covers white matter. N=23 participants.*

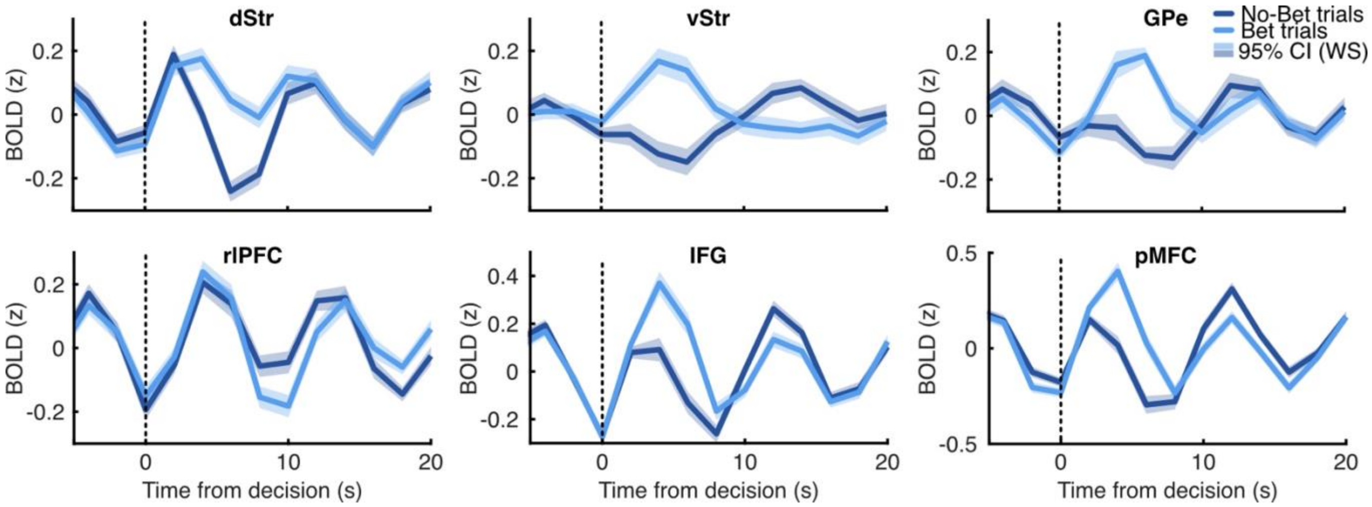

Supplementary Figure S12. *BOLD timeseries of ROIs locked to time from the decision for trials on which the participant bet (light blue) and did not bet (dark blue) separately. These ROIs show a positive relation to post-decision confidence, based on the EEG bet-prediction, used as a regressor in the time-window immediately following the perceptual decision. In the previous literature, these ROIs often show a negative relation to confidence, but the regressor is placed in the later time-window corresponding to the confidence report. The BOLD timeseries shows this is consistent with our results, where the relation to confidence later flips. Abbreviations: dStr, dorsal striatum; vStr, ventral striatum; GPe, external globus pallidus; rlPFC, rostrolateral prefrontal cortex; IFG, inferior frontal gyrus; pMFC, posterior medial frontal cortex. N=23 participants.*

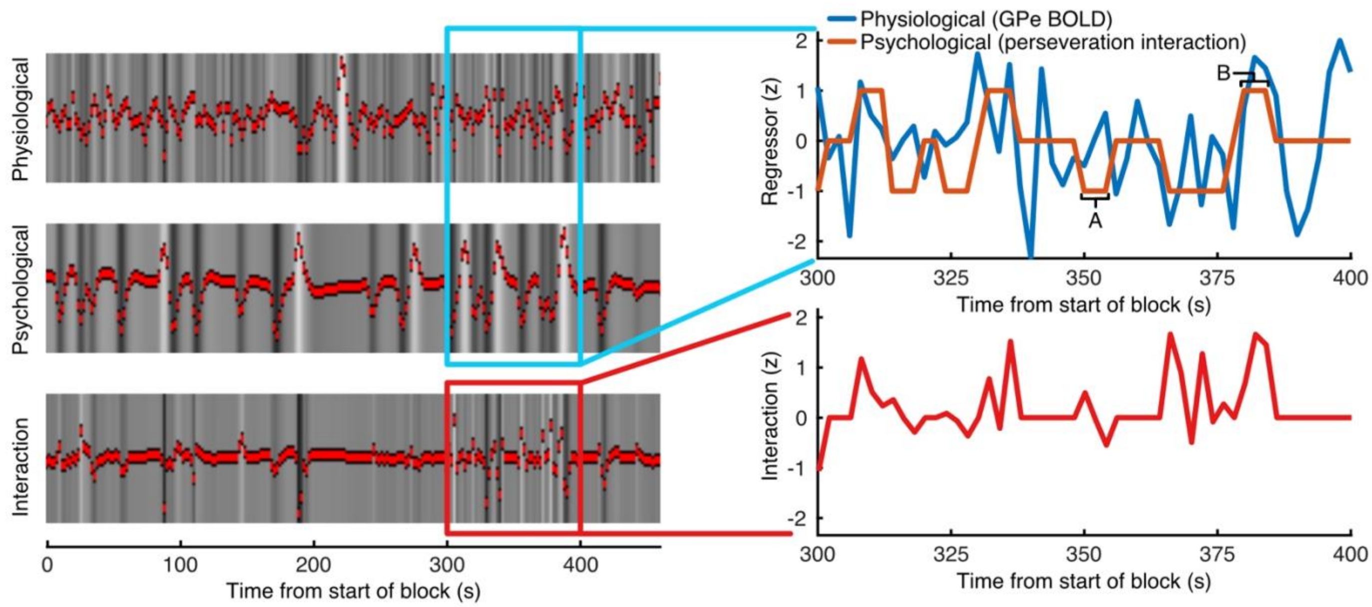

Supplementary Figure S13. Psychophysiological interaction analysis design. *The images on the right show the resulting GLM regressors for one block from one subject. The physiological variable (top) is the GPe BOLD timeseries, taken as the average across voxels in the cluster. The psychological variable (middle) is the perseveration interaction, which is zero except in the time window from feedback over the intertrial interval, where it is set to 1 when the participant behaves according to learning from feedback (alternating responses to repeating stimuli following negative feedback (or no-bet responses on no-feedback trials), as well as trials with repeating responses to repeating stimuli following positive feedback (or bet responses on no-feedback trials), or -1 otherwise (repeating a response for a repeated stimulus following negative feedback (or no-bet trials), as well as alternating responses to repeating stimuli following positive feedback (or bet trials). The interaction is the variable of interest, where clusters of BOLD associated with this variable show increased connectivity with GPe when the participant's next response will be in line with learning from feedback (or decreased connectivity when the next response is not in line with learning from feedback). The panels to the right zoom in on the variables within the 300-400 s time-window. As a demonstration, the part of the psychological variable marked as A corresponds to the feedback window on trial 41 wherein the participant received negative feedback and on trial 42 the same stimulus direction was repeated and the participant repeated their response. The part of the psychological variable marked as B corresponds to the feedback window on trial 43 wherein the participant received negative feedback and on trial 44 the same stimulus direction was repeated but the participant changed their response.*

PPI: All trials

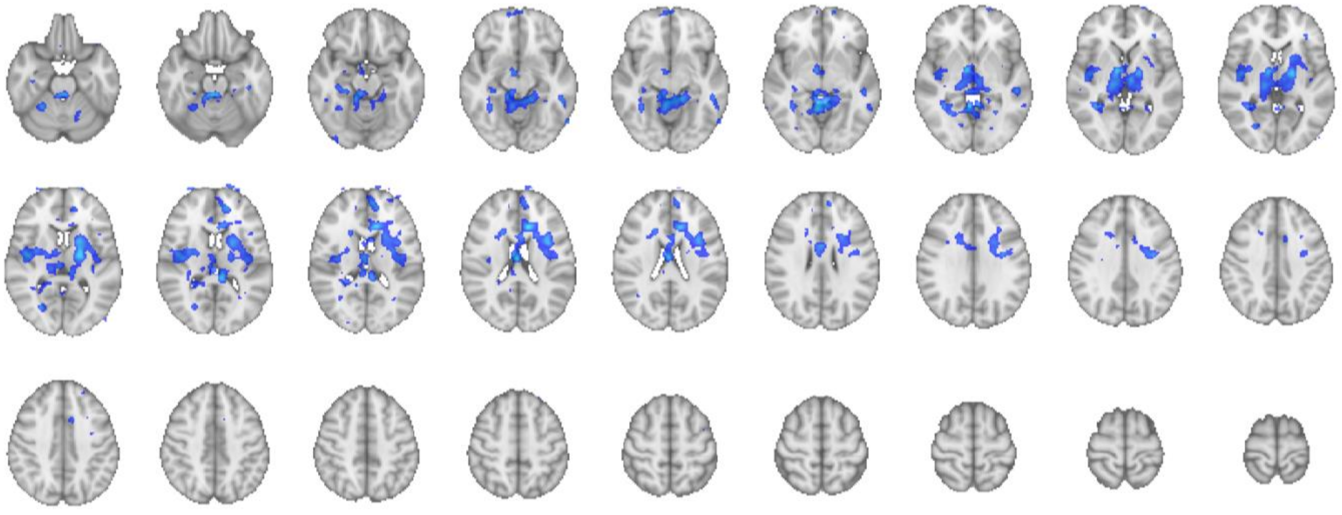

PPI: All trials vs. explicit-feedback trials

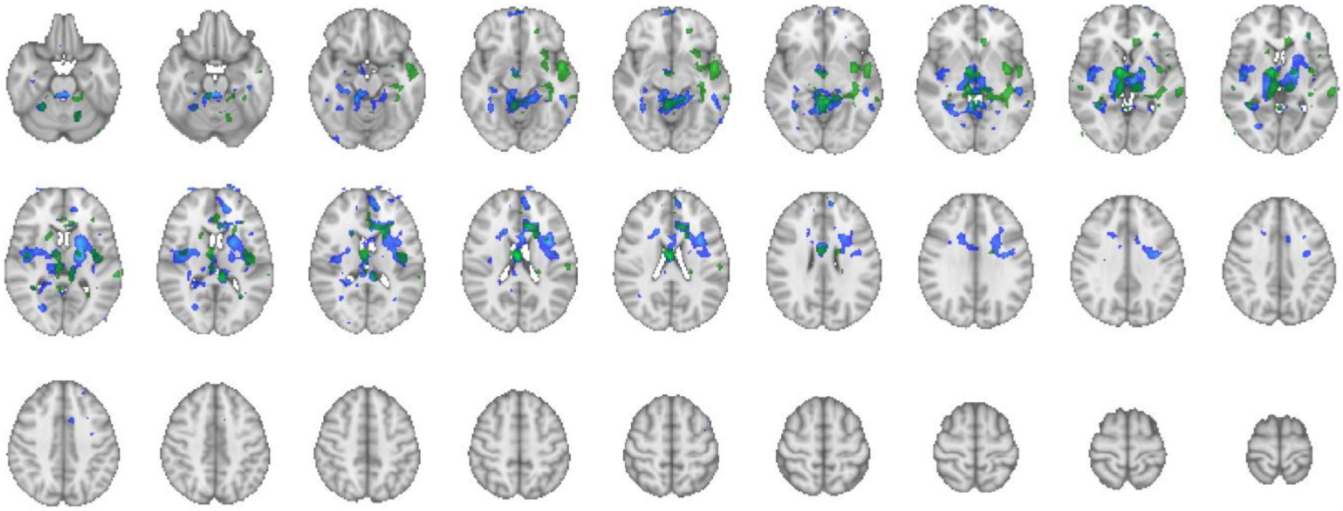

PPI: All trials vs. no-feedback trials

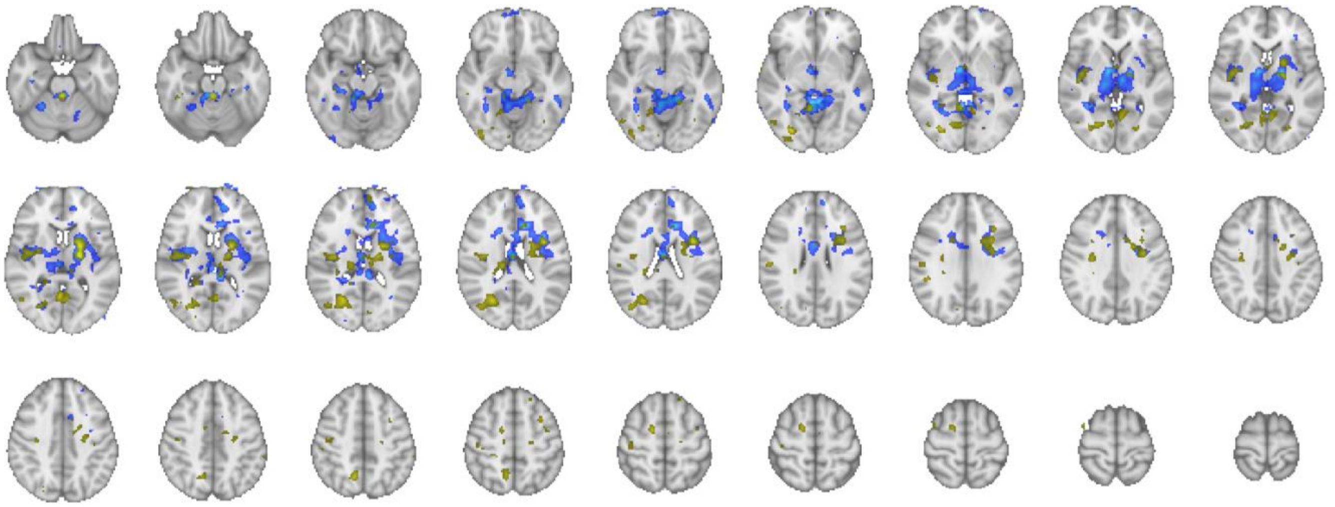

Supplementary Figure S14. *Psychophysiological interaction Z-statistics for the analysis including all trials (top) compared to explicit-feedback trials (middle) and no-feedback trials (bottom). Coloured regions highlight Z >= 2.57 for the interaction between the BOLD time-course from the external globus pallidus region of interest and a variable coding the interaction between response perseveration and feedback (positive for repeating response following positive feedback and alternating response following negative feedback for repeating stimuli, the opposite combination for alternating stimuli, and otherwise negative). N=23 participants.*

Post-decision confidence					
**Label**	**Voxels**	**MAX (Z-stat)**	**MAX X (MNI)**	**MAX Y (MNI)**	**MAX Z (MNI)**
Left Motor/Somatosensory cortex including posterior middle frontal	4407	5.72	-34	-24	60
Left inferior frontal gyrus, GPe	1558	4.13	-60	8	22
Right Parietal/somatosensory	577	3.97	54	-20	40
Right inferior fronal gyrus / precentral gyrus	510	4.23	54	4	36
Left occipital	388	4.41	-40	-66	-8
Cerebellum	288	3.63	16	-46	-20
Left rostrolateral PFC	235	3.49	-28	52	18
Right middle frontal gyrus	134	3.46	38	38	30
**Expected outcome value**					
**Label**	**Voxels**	**MAX (Z-stat)**	**MAX X (MNI)**	**MAX Y (MNI)**	**MAX Z (MNI)**
Left occipital	330	3.4	-36	-94	-4
Left parietal	132	3.12	-40	-36	40
Left insular	124	3.79	-50	-20	20
**Explicit outcome value**					
**Label**	**Voxels**	**MAX (Z-stat)**	**MAX X (MNI)**	**MAX Y (MNI)**	**MAX Z (MNI)**
Bilateral striatum, ventromedial and ventrolateral PFC, left Gpe	14915	5.8	-14	-14	-10
Cerebellum	6558	4.71	-6	-82	-28
Right Parietal	1247	4.27	42	-50	48
Left superior parietal	845	3.91	-46	-48	58
Left middle temporal gyrus	567	3.89	-58	-40	-8
Right middle temporal gyrus	234	3.55	54	-48	-8
Left Insular	213	3.77	-42	2	4
Right temporal lobe	188	3.49	56	-24	-20
Bilateral paracingulate gyrus	173	3.36	12	34	32
Right Parietal	156	3.73	14	-66	46
**Implicit outcome value**					
**Label**	**Voxels**	**MAX (Z-stat)**	**MAX X (MNI)**	**MAX Y (MNI)**	**MAX Z (MNI)**
Right occipital	180	3.19	30	-96	4
Left middle frontal gyrus	170	3.05	-36	6	62
Left dorsal striatum	112	3.18	-20	18	6

Supplementary Table S1. *Cluster results for the fMRI GLM analysis (variables of interest only), including 23 participants.*
