# Supplementary material for: Distinct basal ganglia contributions to learning from implicit and explicit value signals in perceptual decision-making
Source: Nat Commun. 2024 Jun 22;15:5317. doi: 10.1038/s41467-024-49538-w (PMC11193814; doi:10.1038/s41467-024-49538-w)
Supplement: Supplementary file 3 — Reporting Summary [file 41467_2024_49538_MOESM3_ESM.pdf]

nature portfolio

Corresponding author(s): Tarryn Balsdon

Last updated by author(s): Apr 30, 2024

Reporting Summary

Nature Portfolio wishes to improve the reproducibility of the work that we publish. This form provides structure for consistency and transparency in reporting. For further information on Nature Portfolio policies, see our Editorial Policies and the Editorial Policy Checklist.

Statistics

For all statistical analyses, confirm that the following items are present in the figure legend, table legend, main text, or Methods section.

n/a	Confirmed	
☐	☒	The exact sample size (n) for each experimental group/condition, given as a discrete number and unit of measurement
☐	☒	A statement on whether measurements were taken from distinct samples or whether the same sample was measured repeatedly
☐	☒	The statistical test(s) used AND whether they are one- or two-sided<br>*Only common tests should be described solely by name; describe more complex techniques in the Methods section.*
☐	☒	A description of all covariates tested
☐	☒	A description of any assumptions or corrections, such as tests of normality and adjustment for multiple comparisons
☐	☒	A full description of the statistical parameters including central tendency (e.g. means) or other basic estimates (e.g. regression coefficient) AND variation (e.g. standard deviation) or associated estimates of uncertainty (e.g. confidence intervals)
☐	☒	For null hypothesis testing, the test statistic (e.g. F, t, r) with confidence intervals, effect sizes, degrees of freedom and P value noted<br>*Give P values as exact values whenever suitable.*
☒	☐	For Bayesian analysis, information on the choice of priors and Markov chain Monte Carlo settings
☒	☐	For hierarchical and complex designs, identification of the appropriate level for tests and full reporting of outcomes
☐	☒	Estimates of effect sizes (e.g. Cohen's d, Pearson's r), indicating how they were calculated

Our web collection on statistics for biologists contains articles on many of the points above.

Software and code

Policy information about availability of computer code

Data collection	Stimulus presentation was controlled with custom code using Presentation software
Data analysis	Data analysis was conducted using FSL software and custom code written in matlab and available on the OSF page: https://osf.io/q29uf/

For manuscripts utilizing custom algorithms or software that are central to the research but not yet described in published literature, software must be made available to editors and reviewers. We strongly encourage code deposition in a community repository (e.g. GitHub). See the Nature Portfolio guidelines for submitting code & software for further information.

Data

Policy information about availability of data

All manuscripts must include a data availability statement. This statement should provide the following information, where applicable:

- Accession codes, unique identifiers, or web links for publicly available datasets
- A description of any restrictions on data availability
- For clinical datasets or third party data, please ensure that the statement adheres to our policy

Raw behavioural data, pre-processed EEG, and fMRI Z-statistic maps are available on the Open Science Framework: https://osf.io/q29uf/

Research involving human participants, their data, or biological material

Policy information about studies with human participants or human data. See also policy information about sex, gender (identity/presentation), and sexual orientation and race, ethnicity and racism.

Reporting on sex and gender	We do not perform analyses based on sex, gender, race, or ethnicity. There are no grounds to hypothesise differences in this context. We report 17/30 participants identified as male.
Reporting on race, ethnicity, or other socially relevant groupings	We report: "Participants (N = 30; 17 male/ 13 female; age range: 22–33 years)" this was self-reported sex, we assume it is obvious that we did not perform a physical check. We do not make any hypotheses about social groupings, nor do we perform any analyses on these potential variables.
Population characteristics	We report 17/30 participants identified as male, we asked that participants have no history of neuropsychiatric diagnosis. This is standard practice at our institution when recruiting participants for fMRI.
Recruitment	Participants were recruited from the psychology subject pool, which is largely comprised of students. The study is advertised to the subject pool using an online platform and participants request to participate. There may have been some self-selection bias, we do not think this would impact the results.
Ethics oversight	College of Science and Engineering Ethics Committee at the University of Glasgow (CSE01355)

Note that full information on the approval of the study protocol must also be provided in the manuscript.

Field-specific reporting

Please select the one below that is the best fit for your research. If you are not sure, read the appropriate sections before making your selection.

☒ Life sciences ☐ Behavioural & social sciences ☐ Ecological, evolutionary & environmental sciences

For a reference copy of the document with all sections, see nature.com/documents/nr-reporting-summary-flat.pdf

Life sciences study design

All studies must disclose on these points even when the disclosure is negative.

Sample size	30 participants were recruited, with 23 included in the analysis (see below). This sample was determined based on previous EEG-fMRI experiments (Arabadzhiyska, D. H. et al. A Common Neural Account for Social and Nonsocial Decisions. J. Neurosci. 42, 9030–9044 (2022).)
Data exclusions	Due to technical problems in collection of simultaneous EEG-fMRI, two participants were excluded, an additional four participants were excluded for performance below 55% correct, and one participant for too few 'bet' responses (betting on fewer than 15% of trials)
Replication	We have not attempted to perform a replication experiment. The collection of simultaneous EEG-fMRI data is expensive and arduous for the participant.
Randomization	All participants performed the experiment as part of the same group (within participants design).
Blinding	There was no group allocation.

Reporting for specific materials, systems and methods

We require information from authors about some types of materials, experimental systems and methods used in many studies. Here, indicate whether each material, system or method listed is relevant to your study. If you are not sure if a list item applies to your research, read the appropriate section before selecting a response.

Materials & experimental systems

n/a	Involved in the study	
☒	☐	Antibodies
☒	☐	Eukaryotic cell lines
☒	☐	Palaeontology and archaeology
☒	☐	Animals and other organisms
☒	☐	Clinical data
☒	☐	Dual use research of concern
☒	☐	Plants

Methods

n/a	Involved in the study	
☒	☐	ChIP-seq
☒	☐	Flow cytometry
☐	☒	MRI-based neuroimaging

Magnetic resonance imaging

Experimental design

Design type	event-related
Design specifications	6 blocks of 50 trials, 235 functional volumes per block, trials separated by variable duration between 2 and 3 seconds
Behavioral performance measures	Correct/incorrect responses, response times, bet decisions. We required performance above 55% correct, and more than 15% of trials with a bet decision.

Acquisition

Imaging type(s)	Functional and structural
Field strength	3T
Sequence & imaging parameters	Functional volumes (235 per block) were captured with a T2*-weighted gradient echo, echo-planar imaging sequence (32 interleaved slices, gap: 0.3 mm, voxel size: 3 x 3 x 3 mm, matrix size: 70 x 70, FOV: 210 mm, TE: 30 ms, TR: 2000 ms, flip angle: 80). An anatomical reference image was acquired using a T1-weighted sequence (192 slices, gap: 0.5 mm, voxel size: 1 x 1 x 1 mm, matrix size: 256 x 256, FOV: 256 mm, TE: 2300 ms, TR: 2.96 ms, flip angle: 9). For distortion correction, phase and magnitude field maps were acquired (3 x 3 x 3 mm voxels, 32 axial slices, TR: 488 ms, short TE: 4.92 ms, long TE: 7.38 ms).
Area of acquisition	Whole brain

Diffusion MRI ☐ Used ☒ Not used

Preprocessing

Preprocessing software	fMRI analyses were conducted using FSL software [67] (FMRIB, fmrib.ox.ac.uk/fsl). The Brain Extraction Tool [68] (BET) was used for brain extraction of the structural images and local field maps. The first 5 volumes of each functional run were discarded. Functional images were slice-time corrected, high-pass filtered at 50 Hz, spatially smoothed (8mm full width half maximum Gaussian kernel), and unwarped using the field maps (using FEAT [69,70]). Motion correction (using MCFLIRT [71]) was performed with parameters saved for later use as nuisance regressors in the GLM analysis. A two-stage registration aligned functional to structural with boundary-based registration, and structural to standard Montreal Neurological Institute (MNI) space with a 12 DOF, nonlinear search. (numbers in square brackets correspond to references in manuscript)
Normalization	Montreal Neurological Institute (MNI) space with a 12 DOF, nonlinear search.
Normalization template	Montreal Neurological Institute (MNI) space with a 12 DOF, nonlinear search.
Noise and artifact removal	Motion correction (using MCFLIRT [71]) was performed with parameters saved for later use as nuisance regressors in the GLM analysis.
Volume censoring	N/A

Statistical modeling & inference

Model type and settings	We used an EEG-informed GLM analysis with eight explanatory variables and seven confound variables (the six motion correction parameters, and a custom variable coding for excluded trials and mid-block breaks). Explanatory variables were modelled as boxcar functions convolved with a normalised double-gamma probability density function.
Effect(s) tested	The eight explanatory variables were temporally positioned: (1) at stimulus onset, (2) the participant-specific time-point selected for the bet-prediction spatial filter relative to response time, (3) the participant specific time-point where the feedback-prediction best generalised relative to response time, (4 and 5) the time of the bet-cue (for bet and no-bet trials separately), (6) locked to the feedback cue, (7) the participant-specific time-point selected for the feedback-prediction spatial filter relative to feedback time, and (8) the participant specific time-point where the bet-prediction best generalised relative to feedback. All variables were a boxcar function with a duration of 0.1 s, with the exception of the bet cue on bet trials (4; which was extended to the time of the bet-response), and the feedback cue (6; 1 s, the duration of the cue). The boxcar amplitude of the bet-cue on bet trials, the bet-cue on no-bet trials and the feedback-cue variables (4, 5, and 6) was set to 1. The boxcar amplitude of the stimulus onset variable (1) was modulated by the choice reaction time on each trial. The boxcar amplitudes for the remaining four variables were parametrically modulated by the EEG predictions (the bet-prediction in the decision-window, (2); the feedback-prediction in the decision-window, (3); the feedback-prediction in the feedback-window, (7); and the bet-prediction in the feedback-window (8)). These variables correspond to EEG-predictors 1-4 in Figure 2C.

Specify type of analysis: ☐ Whole brain ☐ ROI-based ☒ Both

Anatomical location(s): ROIs were defined post-hoc from the whole-brain analysis

Statistic type for inference

(See Eklund et al. 2016)

Significant clusters were selected using a minimum z-statistic of 2.57 at the group level, and a minimum cluster size of 110 voxels, obtained using a permutation test (described previously [37,64]; the 95th percentile of the empirical null cluster size, calculated over 200 permutations of the EEG feedback-prediction variable (7) order with otherwise the same GLM described above).

Correction

Minimum cluster size based on permutations test

Models & analysis

n/a	Involved in the study
☐	☒ Functional and/or effective connectivity
☒	☐ Graph analysis
☐	☒ Multivariate modeling or predictive analysis

Functional and/or effective connectivity

We used vector autoregressive models of the full timeseries of GPe and IFG BOLD to test for Granger causality. For each participant, we first selected the appropriate lag in the model based on the Akaike Information Criterion (AIC). The median lag was 6 (12 s) ranging from 2 – 17 (4 – 34 s). All models were found to be stable. Leave-one-out Granger causal tests were conducted within subjects, with 18/23 participants showing significant evidence against the null hypothesis to exclude the history of IFG BOLD in predicting GPe BOLD (over and above the history of GPe BOLD itself).

Multivariate modeling and predictive analysis

See above description of GLM analysis. We also conducted a Psychophysiological Interaction (PPI) analysis [79] to examine how GPe could be used to implement learning. As the physiological variable we took the time-course of the voxel average BOLD in the GPe ROI from the time of feedback to the start of the next trial. The psychological variable described the interaction between stimulus and response repetition that we took as a behavioural signature of learning: trials were coded as +1 for alternating responses to repeating stimuli following negative feedback (or no-bet responses on no-feedback trials), as well as trials with repeating responses to repeating stimuli following positive feedback (or bet responses on no-feedback trials); trials were coded as -1 for repeating a response for a repeated stimulus following negative feedback (or no-bet trials), as well as alternating responses to repeating stimuli following positive feedback (or bet trials). This can be summarised as a positive coding for trials contributing to the positive bars in Figure 1F and a negative coding for trials contributing to the negative bars in Figure 1F. For further demonstration, an example of the psychological, physiological, and interaction regressors are visually displayed with some annotation in Supplementary Figure 11. Clusters of significant voxels were again selected with a statistical threshold of z >= 2.57 and minimum size of 110 voxels.
